# Metabolomic Analysis of Wheat Grains after *Tilletia laevis* Kühn Infection by Using Ultrahigh-Performance Liquid Chromatography–Q-Exactive Mass Spectrometry

**DOI:** 10.3390/metabo12090805

**Published:** 2022-08-28

**Authors:** Muhammad Jabran, Delai Chen, Ghulam Muhae-Ud-Din, Taiguo Liu, Wanquan Chen, Changzhong Liu, Li Gao

**Affiliations:** 1 State Key Laboratory for Biology of Plant Disease and Insect Pests, Institute of Plant Protection, Chinese Academy of Agricultural Sciences, Beijing 100193, China; 2 College of Life Science and Technology, Longdong University, Qingyang 745000, China; 3 College of Plant Protection, Gansu Agricultural University, Lanzhou 730070, China

**Keywords:** *Tilletia laevis*, suspension, extensively targeted metabolomics, differential metabolites, metabolic pathway

## Abstract

*Tilletia laevis* causes common bunt disease in wheat, with severe losses of production yield and seed quality. Metabolomics studies provide detailed information about the biochemical changes at the cell and tissue level of the plants. Ultrahigh-performance liquid chromatography–Q-exactive mass spectrometry (UPLC-QE-MS) was used to examine the changes in wheat grains after *T. laevis* infection. PCA analysis suggested that *T. laevis*-infected and non-infected samples were scattered separately during the interaction. In total, 224 organic acids and their derivatives, 170 organoheterocyclic compounds, 128 lipids and lipid-like molecules, 85 organic nitrogen compounds, 64 benzenoids, 31 phenylpropanoids and polyketides, 21 nucleosides, nucleotides, their analogues, and 10 alkaloids and derivatives were altered in hyphal-infected grains. According to The Kyoto Encyclopedia of Genes and genomes analysis, the protein digestion and absorption, biosynthesis of amino acids, arginine and proline metabolism, vitamin digestion and absorption, and glycine, serine, and threonine metabolism pathways were activated in wheat crops after *T. laevis* infection.

## 1. Introduction

Common bunt disease of wheat caused by *T. laevis* Kühn (syn. *T. foetida* (Wallr.) Liro.) is one of the most serious fungal diseases affecting wheat crops globally, especially in China and United State of America [1,2]. *T. laevis*, belongs to the phylum *Basidiomycota* within the kingdom Fungi, which includes many destructive pathogens of plants [3]. Plants infected with *T. laevis* generally produce a low grain yield with poor quality compared with un-infected plants [4,5]. The reduction in the yield and its quality in *T. laevis*-infected plants occurs due to the replacement of kernels with bunt balls of teliospores [6,7]. Furthermore, wheat millers generally reject grains infected by *T. laevis,* as very low infection rates can result in noticeable undesirable odors in flour [4]. The germination of the *T. laevis* teliospores coincides with the germination of the wheat seedlings shortly after seeding; teliospore germination is usually completed 4–10 days after seeding [8,9] and is followed by the penetration of emerging wheat coleoptiles 7–10 days post-inoculation [10]. A successful infection results when *T. laevis* is able to establish itself in the region directly below the growing point of the developing seeding, which occurs 2–5 weeks after seeding [11,12,13]. According to our knowledge, clear evidence of a hypersensitive reaction has been observed in bunt–wheat interactions [13].

Plants have an ability to produce thousands of unique metabolites that serve to attract pollinators, combat microbial pathogens, repel herbivores, and also act against abiotic stress [14]. Plant metabolism is generally divided into two categories: primary and specialized metabolism [15]. Primary metabolites involve compounds for the development, growth, and reproduction of the plants, while specialized metabolites include compounds needed for plants to protect themselves from biotic and abiotic stress [15,16,17]. These classes of metabolism are intrinsically linked; the metabolites of the primary metabolic pathways, such as the pentose–phosphate pathway, glycolysis, and the tricarboxylic acid cycle, also serve as building blocks for secondary metabolic pathways. Amino acids, for example, not only play a role in nitrogen assimilation, but they also act as precursors for a number of specialized compounds, including pigments and hormones [18]. The plant–pathogen interaction studies that integrated observations of host metabolism have paved the way to a better understanding of plant disease mechanisms [19,20,21,22,23].

As a new technology, metabolomics is extensively applied in primary and secondary metabolite identification, and analyzing response mechanisms, microbial interactions, and gene function [24,25]. Keon et al. [26] used proton nuclear magnetic resonance to study the nutrient content of the apoplast of wheat leaves after infection by *Mycosphaerella graminicola*. Transcriptomics and metabolomics have a role in studying the regulation of primary metabolism, antioxidants, and stress tolerance in soybean after *Rhizoctonia* foliar blight disease [27]. Song et al. [28] used a liquid culture of *Aspergillus flavus* to study the metabolic pathways by using gas chromatography–mass spectrometry, and 1181 and 490 volatile substances were identified and separated, respectively, and 332 were found intracellularly. Here, we used UHPLC-QE-MS to identify the types, changes, and metabolic pathways of various metabolites in *Tilletia laevis* hyphae development. According to our knowledge, this is the first study to report the metabolic changes in *T. laevis* hyphae development.

## 2. Materials and Methods

### 2.1. Fungal Strain and Spore Suspension

*T. laevis* was isolated from infected wheat grains at the Institute of Plant Protection, Chinese Academy of Agricultural Sciences. *T. laevis*-infected grains were stored in 2 mL centrifuge tubes overnight, sterilized by adding 0.25% NaClO solution for 1 min, washed 3 times with ddH_2_O, and cultured on agar medium in an incubator (MLR 352 H, Panasonic, Japan) for 6 days. The density of the teliospores was adjusted to 1 × 10^6^/mL. The hyphae of *T. laevis* were collected and transferred into sterile water, and centrifuged at 150 rpm/min and 16 °C for 3 days. The germination of teliospores was checked using an automated inverted fluorescence microscope (IX83, Olympus, Tokyo, Japan). The teliospores were characterized into five categories based on growth: suspensions of teliospores (T1), promycelia (T2), primary basidiospores (T3), H-bodies (T4), and secondary basidiospores (T5). Each suspension was centrifuged to collect the supernatant. Samples of every treatment were stored at −80 °C for further use. Sterilized soil extracts were used as the control group.

### 2.2. Metabolites Extraction for Liquid Chromatography-Mass Spectrometry (LC-MS) Analysis

The metabolites were extracted by following the method of previous reports [29,30]. An amount of 30 mg of infected grains was added to a 2 mL Eppendorf tube; 500 µL of extract solvent (acetonitrile:methanol:water: 2:2:1) was added and the samples were ground for 2 min. The above samples were incubated at −20 °C for 1 h and centrifuged at 13,000 rpm for 15 min at 4 °C. An amount of 350 µL of supernatant was transferred to a 1.5 mL Eppendorf tube and dried in a vacuum concentrator for the next step. The metabolites were dissolved again in extract solvent (acetonitrile: water: 1:1), vortexed for 30 s, and centrifuged at 13,000 rpm for a further 15 min at 4 °C. An amount of 50 µL of supernatant was transferred to LC vials, followed by injection in the LC-MS system.

### 2.3. Ultra-High-Performance Liquid Chromatography UHPLC-QE-MS Analysis

All LC-MS analyses were carried out using the same UHPLC system: ACQUITY UPLC BEH Amide (Waters, Milford, MA, USA). Screening a large set of conditions was the first step of method optimization, comprising a binary pump, an autosampler of the fixed loop type with a 10 µL sample loop, and a column thermostat. MS/MS detection was accomplished using a quadrupole time-of-flight mass spectrometer (QTOF MS; Triple TOF 5600+, SCIEX, Framingham, MA, USA) system with an electrospray ionization (ESI) source operated in the positive mode. The MS ion source conditions were as follows: ion spray voltage (Ion Spray Voltage Floating) of 5500 V, ion gas temperature of 650 °C, ion gas pressure of 60 psi, curtain gas at 30 psi, declustering potential of 60 V, and collision energy of 10 eV.

Mobile phase A contained ammonium acetate and ammonium hydroxide (25 mM:25 mM) in water, while mobile phase B contained 100% ACN. The gradient program was 95–65% B for 0–7 min, 65–40% B for 7–8 min, 40% B for 8–9 min, 40–95% B for 9–9.1 min, and 95% B for 9.1–12 min with a flow rate of 0.5 mL/min. The samples were stored in an autosampler at a temperature of 4 °C.

### 2.4. Data Analysis

All UHPLC-QE-MS data was further filtered by using the R platform loaded with the xcms tool kit, including peak matching, retention time correction, variable integration (to integrate the overall contribution of each variable), and data standardization (non-target metabolites’ data processing). The raw data were preprocessed by noise reduction, baseline correction, peak alignment, standardization, and scaling, and then analyzed by multivariate analysis, including principal components analysis (PCA), orthogonal partial-least-squares discriminant analysis (OPLS-DA) with EZinfo software, and hierarchical cluster analysis (HCA) using MetaboAnalyst 4.0 software (https://www.metaboanalyst.ca/, accessed on 1 July 2022). Based on the OPLS-DA, a loading plot was constructed to determine the variable importance in the projection (VIP). VIP values exceeding 1.0 were selected to indicate changed metabolites, while higher values were assessed by Student’s *t*-test (Q-value > 0.05) for further analysis. The metabolic pathways were further analyzed using KEGG (http://www.genome.jp/kegg/, accessed on 1 July 2022). The correlation analysis of differential metabolites was calculated using R, and Cytoscape software was used for network construction.

## 3. Results

### 3.1. Qualitative and Quantitative Analysis of Metabolites

**Figure 1:**
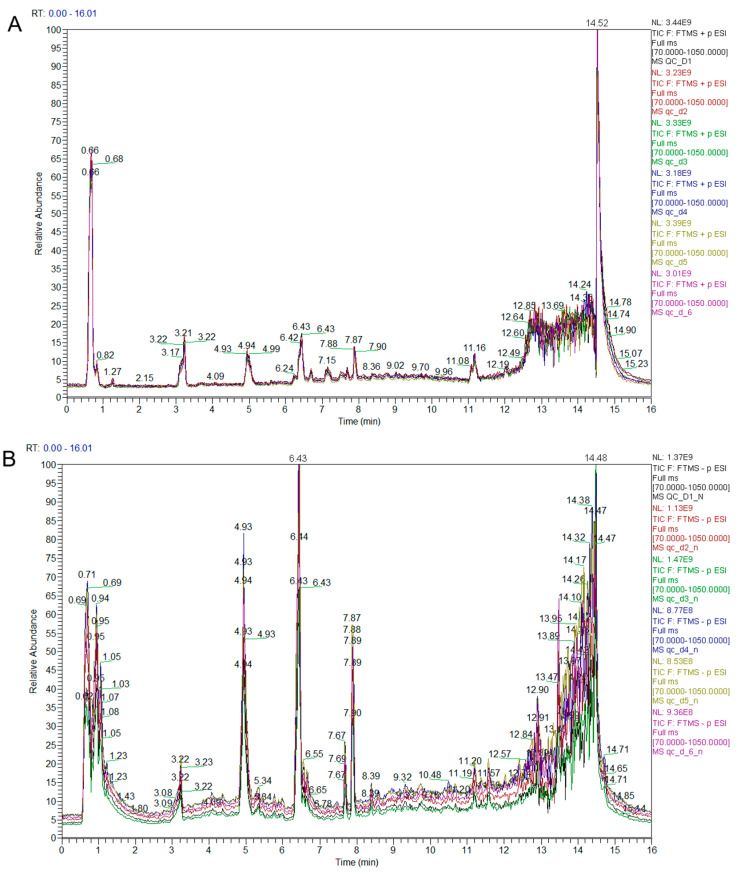
LC–MS chromatograms of the wheat grains’ metabolites (*X*-axis = time and *Y*-axis = response. (**A**) Relative abundance of positive ions at different time intervals. (**B**) Relative abundance of negative ions at different time intervals.

The results showed that the retention time and peak intensities of the QC samples were consistent, the curves overlapped, and the signal stability was good, which provides a guarantee of the reproducibility and reliability of the data (Figure 1A,B). The metabolite detection and analysis were conducted in both the positive- and negative-ion modes, and the total ion currents (TICs) of mixed quality-control (QC) samples were overlaid to obtain the positive-ion TIC overlay (Figure 1A) and negative-ion TIC overlay (Figure 1B). The peak values of the positive and negative ions were obtained within 0–16 min, as shown in Figure 1a,b. Additionally, 734 metabolites were detected, which were divided into eight types: organic acids and derivatives (30.52%); organoheterocyclic compounds (23.16%); lipids and lipid-like molecules (17.44%); organic nitrogen compounds (11.58%); benzenoids (8.71%); phenylpropanoids and polyketides (4.35%); nucleosides, nucleotides, and their analogues (2.86%); and alkaloids and their derivatives (1.36%) (Table 1).

### 3.2. PCA and Orthogonal Projections to Latent Structures Discriminant Analysis

**Figure 2:**
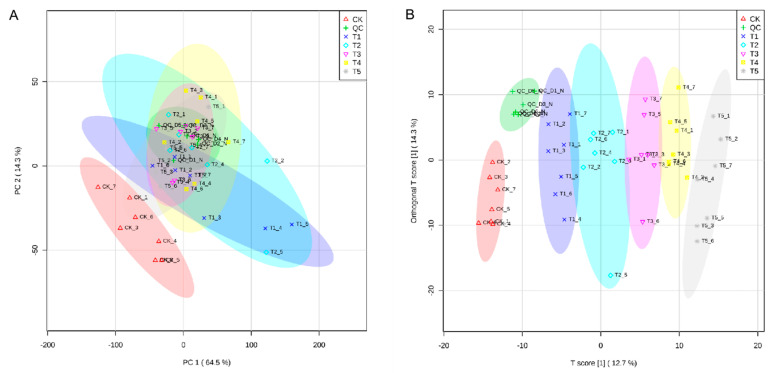
PCA and OPLS-DA of *T. laevis*-infected and control grains. (**A**) PCA of grains during different fungal hyphal growth. (**B**) Orthogonal partial least squares discrimination analysis (OPLS-DA) (Q = 0.938). CK stands for control, QC stands for quality control, T1 stands for suspension of teliospores, T2 stands for promycelia, T3 stands for primary basidiospores, T4 stands for H-bodies, and T5 stands for secondary basidiospores.

**Figure 3:**
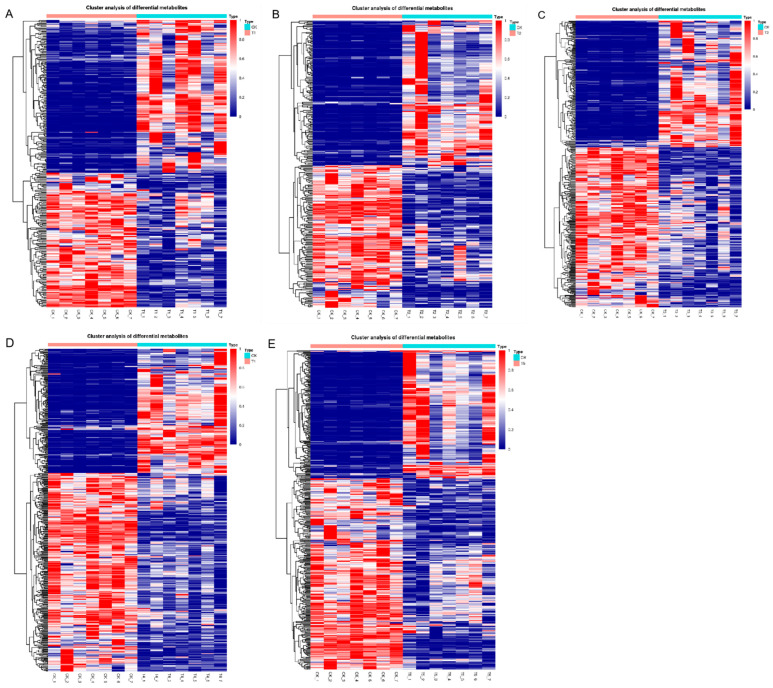
Hierarchical clustering heatmap visualizing the changes in the contents of potential metabolites in grains after different hyphal development stages in *Tilletia laevis* infection. (**A**) CK vs. T1 group. (**B**) CK vs. T2 group. (**C**) CK vs. T3 group. (**D**) CK vs. T4 group. (**E**) CK vs. T5 group. CK stands for control, QC stands for quality control, T1 stands for suspension of teliospores, T2 stands for promycelia, T3 stands for primary basidiospores, T4 stands for H-bodies, and T5 stands for secondary basidiospores.

As shown in Figure 2A, the results of principal component analysis (PCA) showed obvious separation between the groups, suggesting that all different samples clustered separately, suggesting that they had specific metabolic profiles under *T. laevis* infection in comparison with the wild-type (PC1, 64.5%), as well as to each other (PC2, 14.3%). To identify the different metabolites with more accuracy, the metabolites were further analyzed based on the orthogonal projections to latent structures discriminant analysis (OPLS-DA) method. The OPLS-DA plot showed that both *T. laevis*-infected and control samples had a clear difference, but there was no difference between the same samples (Figure 2B). Additionally, based on the cluster heat map analysis, the five groups of samples were significantly different, while the parallel samples in each group were close in terms of substrates, demonstrating the reliability of the samples (Figure 3A–E).

### 3.3. Screening and Analysis of Differential Metabolites

**Figure 4:**
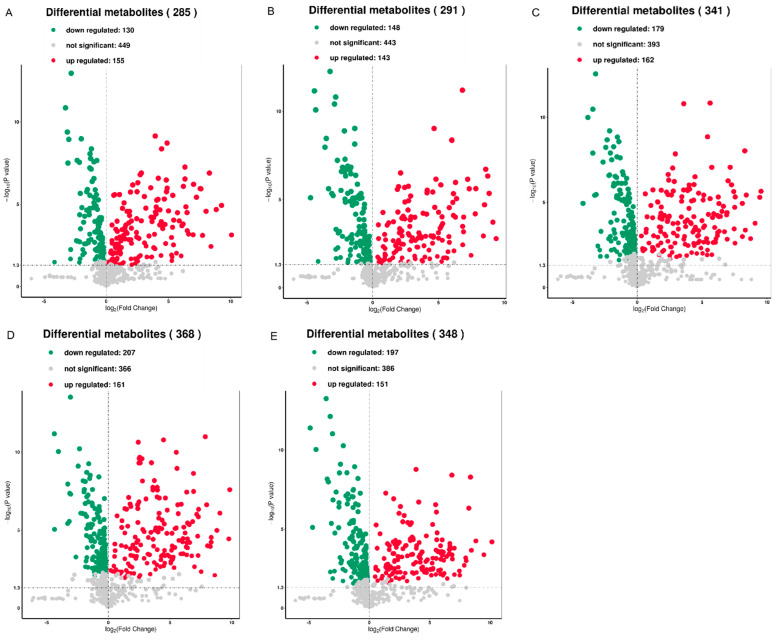
Different metabolites in the grains after different hyphal development stages of *Tilletia laevis* infection. (**A**) Volcano plots of different metabolites in the suspension of teliospores (T1) and the control (CK) group. (**B**) Volcano plots of different metabolites in the promycelia (T2) and control (CK) group. (**C**) Volcano plots of different metabolites in the primary basidiospores (T3) and control (CK) group. (**D**) Volcano plots of different metabolites in the H-bodies (T4) and control (CK) group. (**E**) Volcano plots of different metabolites in the secondary basidiospores (T5) and control (CK) group.

Based on OPLS-DA, metabolites were selected according to the following criteria: fold change of >2 indicating up-regulation and <0.5 indicating down-regulation of various metabolites. Metabolites with a *p*-value of ≤0.5 and VIP of ≥1 in each comparison were selected as significantly changed metabolites. The T1 vs. CK, T2 vs. CK, T3 vs. CK, T4 vs. CK, and T5 vs. CK relationships were visualized using volcano plots according to the log_10_ of the *p*-value (*y*-axis) and log_2_ of the fold-change (*x*-axis) (Figure 4A–E). In the T1 vs. CK relationship, we identified a total of 285 metabolites, out of which 130 were down-regulated and 155 were up-regulated (Figure 4A). In the T2 vs. CK relationship, 291 metabolites were identified—148 were downregulated and 143 were up-regulated (Figure 4B). In the T3 vs. CK relationship, 179 out of 341 different metabolites were down-regulated and 162 were up-regulated (Figure 4C). In the T4 vs. CK relationship, 207 out of 368 metabolites were down-regulated and 161 were up-regulated (Figure 4D). Similarly, in the T5 vs. CK relationship, 197 out of 348 metabolites were down-regulated and 151 were up-regulated (Figure 4E).

### 3.4. Analysis of Metabolic Pathways after T. laevis Infection

**Figure 5:**
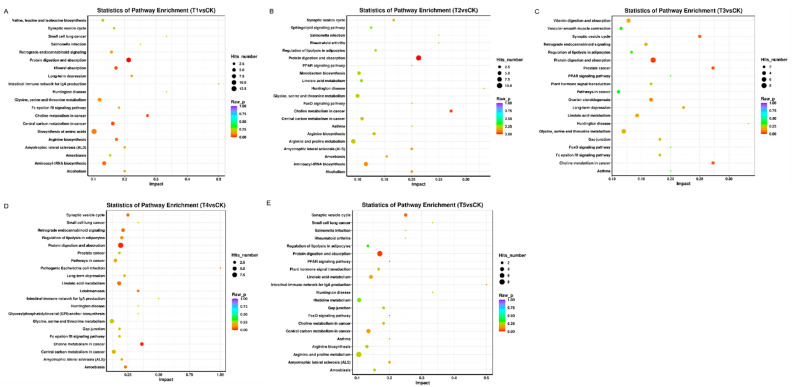
KEGG enrichment analysis scatter plot representing the pathways of DEGs in response to different fungal developmental stages of *T. laevis* infection. The blue, white, and red colors indicate low, medium, and high expression patterns of genes, respectively. (**A**) CK vs. T1 group. (**B**) CK vs. T2 group. (**C**) CK vs. T3 group. (**D**) CK vs. T4 group. (**E**) CK vs. T5 group. CK stands for control, QC stands for quality control, T1 stands for the suspension of teliospores, T2 stands for promycelia, T3 stands for primary basidiospores, T4 stands for H-bodies, and T5 stands for secondary basidiospores.

Kyoto Encyclopedia of Genes and Genomes (KEGG) pathway mapping was also carried out based on orthology (KO) terms for assignments; the results showed that 147, 77, 72, 36, and 40 KEGG pathways were significantly enriched in T1 vs. CK, T2 vs. CK, T3 vs. CK, T4 vs. CK, and T5 vs. CK, respectively. The top 20 KEGG pathways in the T1 vs. CK relationship are shown in Figure 5A, and protein digestion and absorption, and the biosynthesis of amino acids were the top pathways. The top 20 KEGG pathways in T2 vs. CK are shown in Figure 5B—protein digestion and absorption, arginine and proline metabolism, and aminoacyl-tRNA biosynthesis were the top metabolic pathways. Similarly, the top 20 KEGG pathways in T3 vs. CK are shown in Figure 5C, and the top pathways were vitamin digestion and absorption, protein digestion and absorption, and glycine, serine, and threonine metabolism. The top 20 KEGG pathways in T4 vs. CK are shown in Figure 5D, and protein digestion and absorption; linoleic acid metabolism; glycine, serine, and threonine; choline metabolism in cancer; central carbon metabolism in cancer; and amoebiasis were the top pathways. Additionally, the top 20 KEGG pathways in T5 vs. CK are shown in Figure 5E, and protein digestion and absorption, central carbon metabolism in cancer, and arginine and proline metabolism were the top metabolic pathways.

## 4. Discussion

Common bunt and other bunt-related pathogens have long infection times and sparse development in the seedling stage of wheat during early pathogen infection [8]. In fact, the fungal hyphae that establish in infected seedlings remain scarce until a massive proliferation of the fungal hyphae occurs in the vegetative and reproductive tissues of the crop [5,31]. These proliferations of fungal hyphae with different tissues alter the metabolism process in the vegetative and reproductive parts of wheat. In this study, we used suspensions of *T. laevis* as the material to analyze the changes in metabolites at the five different developmental stages of hyphae using UHPLC-QE-MS. Metabolomics can provide a snapshot of plant metabolism during growth and development, and in response to both biotic and abiotic stresses, including nutritional and environmental stresses [32,33,34,35,36]. After the successful establishment of plant–pathogen interactions, pathogens highly depend on host metabolism and, as a result, the metabolism of the host and pathogen become strongly interlinked [37]. It has been revealed that many fungal and bacterial pathogens are able to change host metabolism, e.g., by producing cell wall invertase and sucrolytic enzymes to change the infected tissue into a carbohydrate sink that delivers hexoses and other sugar molecules to the pathogen [38]. Previous studies investigated pathogen metabolism in hosts [39,40]. Our results revealed that 734 metabolites were detected in the different hyphae development stages of *T. laevis*. The following metabolites were included: organic acids and their derivatives; organoheterocyclic compounds; lipids and lipid-like molecules, organic nitrogen compounds, benzenoids, phenylpropanoids, polyketides, nucleosides, nucleotides, their analogues, alkaloids, and their derivatives (Table 1). The organic acid in the higher plants plays a role in the respiration process to resist biotic and abiotic stresses [41], while organoheterocyclic compounds resist wheat *Fusarium* head blight disease [42]. Similarly, nitrogen compounds and phenylpropanoids are basic elements of the carbohydrates with resistance against different plant pathogens [43]. Additionally, different pathways were identified, including protein digestion and absorption, biosynthesis of amino acids, central carbon metabolism in cancer, mineral absorption, and phenylalanine, and tyrosine and tryptophan biosynthesis. These pathways are key regulators that can contribute to the metabolism of the crop.

According to the KEGG enrichment analysis, most metabolites were characterized as being involved in protein digestion and absorption, and the biosynthesis of amino acids in all interactions of fungal stages and plants (Figure 5A–E). Additionally, L-glutamic acid, L-serine, L-phenylalanine, and other amino acids and their derivatives were enriched in the protein digestion and absorption, mineral absorption, and central carbon metabolism in cancer pathways. Phenylalanine metabolism affects the formation and deposition of lignin, which helps plants to improve their immunity against fungal pathogens [44,45]. Phenylaninammo-nialyase (PAL) is an enzyme related to wheat lignification, which participates in phenylalanine metabolism by decomposing L-phenylalanine and provides precursors for lignin biosynthesis. Phenylacetylglycine is consistently up-regulated in liquid cultures of *A. flavus* [28]. Previous studies showed that the role of phenylacetylglycine in *T. laevis* and *A. flavus* is not yet clear, but it has been studied well in penicillin degradation [46]. Phenylpropanoid metabolism is an important pathway of secondary metabolism in organisms [47]. Additionally, the expressions of linoleic acid, (+/−) 9-HODE, and 4-hydroxyphenylacetic acid changed after *T. laevis* infection. (+/−) 9-HODE plays a role in plant growth and enhances the defense response against various diseases [48]. 2,4-Diacetylphloroglucinol plays a role in disease resistance by inducing the roots to secrete amino acid molecules [49]. Similarly, several benzenoid compounds, which originate from trans-cinnamic acid (CA) as a general phenylpropanoid pathway and lack three carbons, are also volatiles. These volatile benzenoid compounds are important components of plant metabolism [50]. In the present study, the expression of 2,4-diacetylphloroglucinol was down-regulated after *T. laevis* infection. Additionally, the relative abundances of metabolites in pathogen-infected and control groups scattered separately, which may suggest that the grains’ metabolites were affected by *T. laevis*. For example, the metabolites that decreased or increased after pathogen infection were involved in energy metabolism. These changes in plant metabolites may have been the result of *T. laevis* infection.

## Figures and Tables

**Table 1:** List of various types of metabolites of *Tilletia laevis* Kühn.

Types	Counts	Percentage (%)
Organic acids and derivatives	224	30.52
Organoheterocyclic compounds	170	23.16
Lipids and lipid-like molecules	128	17.44
Organic nitrogen compounds	85	11.58
Benzenoids	64	8.71
Phenylpropanoids and polyketides	32	4.35
Nucleosides, nucleotides, and their analogues	21	2.86
Alkaloids and their derivatives	10	1.36

## Data Availability

The sequencing data is available at the following link: http://www.genome.jp/kegg/ (accessed on 1 July 2022).
